# Calibration Method of Accelerometer Based on Rotation Principle Using Double Turntable Centrifuge

**DOI:** 10.3390/mi13010062

**Published:** 2021-12-30

**Authors:** Xianshan Dong, Xinlong Huang, Guizhen Du, Qinwen Huang, Yixiong Huang, Yun Huang, Ping Lai

**Affiliations:** 1 Science and Technology on Reliability Physics and Application of Electronic Component Laboratory, No. 5 Electronics Research Institute of the Ministry of Industry and Information Technology, Guangzhou 511370, China; 203712184@csu.edu.cn (X.H.); dgz_xs@163.com (G.D.); huangqinwen@ceprei.com (Q.H.); hyx18819879955@163.com (Y.H.); huangyun@ceprei.com (Y.H.); laiping@ceprei.com (P.L.); 2 College of Mechanical and Electrical Engineering, Central South University, Changsha 410083, China; 3 Institute of Advanced Wear & Corrosion Resistant and Functional Materials, Jinan University, Guangzhou 510632, China

**Keywords:** accelerometer, calibration, double turntable centrifuge, rotation method, installation error

## Abstract

For linear accelerometers, calibration with a precision centrifuge is a key technology, and the input acceleration imposed on the accelerometer should be accurately obtained in the calibration. However, there are often errors in the installation of sample that make the calibration inaccurate. To solve installation errors and obtain the input acceleration in the calibration of the accelerometer, a calibration method based on the rotation principle using a double turntable centrifuge is proposed in this work. The key operation is that the sub-turntable is rotated to make the input axis of the accelerometer perpendicular to the direction of the centripetal acceleration vector. Models of installation errors of angle and radius were built. Based on these models, the static radius and input acceleration can be obtained accurately, and the calibration of the scale factor, nonlinearity and asymmetry can be implemented. Using this method, measurements of the MEMS accelerometer with a range of ±30 g were carried out. The results show that the discrepancy of performance obtained from different installation positions was smaller than 100 ppm after calibrating the input acceleration. Moreover, the results using this method were consistent with those using the back-calculation method. These results demonstrate that the effectiveness of our proposed method was confirmed. This method can measure the static radius directly eliminating the installation errors of angle and radius, and it simplifies the accelerometer calibration procedure.

## 1. Introduction

Accelerometers are widely used in the consumer electronics, automotive, industry and military fields to measure inclination, acceleration and vibration [1,2]. In testing an accelerometer, calibration is a key technology [3,4]. Researchers have carried out a lot of research on the calibration of accelerometers, such as work on auto-calibration [5], the calibration model [6,7] and calibration accuracy under a gravity field [8,9]. Calibrating the scale factor, asymmetry, nonlinearity and range using a precision centrifuge is an indispensable process for the accelerometer [10,11,12]. The static radius error caused by installation makes it difficult to accurately obtain the acceleration about sensor input axis, which is called input acceleration, that leads to calibration error [13,14,15]. Therefore, obtaining accurate installation and input acceleration are important and difficult issues in the calibration of the accelerometer using a precision centrifuge [16,17].

Currently, the calibration methods for the static radius mainly include multipoint tumble testing under Earth’s gravity [18,19], the elimination method of static radius error term [20,21,22] and a method called back calculation [23]. These methods simplify the model, and the static radius is obtained indirectly. For example, for the back-calculation method, the static radius is calculated based on the scale factor calibrated under Earth’s gravity. This method requires dividing head and precision centrifuge equipment, and the calibration procedure is complicated. Moreover, the calibration accuracy is affected by the repeatability of the scale factor, environmental temperature, thermal field and electromagnetic field.

To solve installation errors in the calibration of the accelerometer using a precision centrifuge, a calibration method based on the rotation principle in a double turntable centrifuge is proposed in this paper. Through rotating the sub-turntable, the input axis of the accelerometer is perpendicular to the direction of the centripetal acceleration vector, and the installation errors of angle and radius can be calculated based on our geometric models. Then, the static radius and input acceleration can be obtained, and the calibration of the scale factor, nonlinearity and asymmetry can be implemented. This method needs only a double turntable centrifuge, simplifying equipment requirements. Moreover, the calibration process of our proposed method requires only one installation, which can improve the calibration accuracy and simplify the accelerometer calibration procedure.

## 2. Mathematical Model Based on Rotation Method in Double Turntable Centrifuge

### 2.1. Principle of Double Turntable Centrifuge

**Figure 1:**
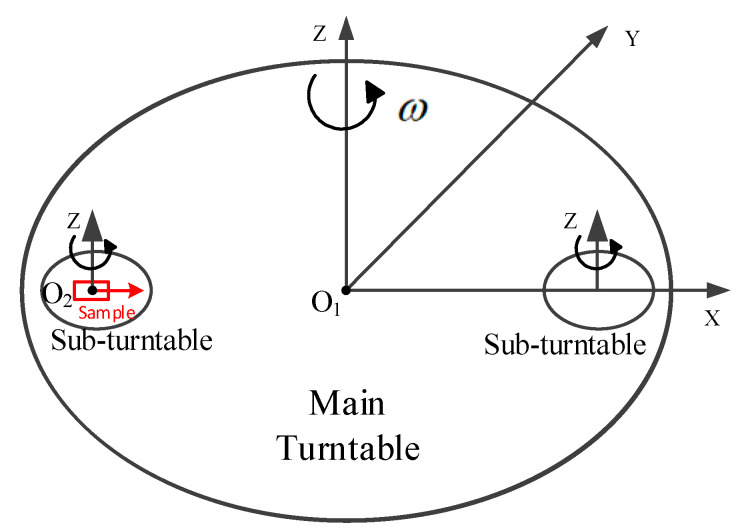
Diagram of working principle for double turntable centrifuge.

The double turntable centrifuge consists of a main turntable and two sub-turntables as shown in Figure 1 where *O*_1_ and *O*_2_ are the rotation centers of the main turntable and sub-turntable, respectively [10,24]. The centripetal acceleration imposed on the accelerometer is generated by the main turntable, and the input axis of the accelerometer can be adjusted by rotating the sub-turntable.

When calibrating aspects such as scale factor and nonlinearity of an accelerometer in a double turntable centrifuge, the sample should be installed on the geometric center of the sub-turntable, and the input axis of sample is collinear with *O*_1_*O*_2_. In the test, the main turntable is rotating, and the sub-turntable is fixed. The centripetal acceleration of centrifuge imposed on the sample is as follows:

(1) $$a=\omega^{2}\times R$$

where *a* is the input acceleration whose unit is m/s^2^, *ω* is the rotation speed of the main turntable whose unit is rad/s and *R* is the static radius whose unit is m. For our double turntable centrifuge, the static radius of the double turntable centrifuge is *R* = 0.4 m, which is the distance between *O*_1_ and *O*_2_.

### 2.2. Errors of Calibration

In the actual test, there may be installation errors that affect calibration accuracy. One is that the sensitive center of the measured accelerometer cannot be completely coincident with the rotation center of the sub-turntable. Another is that there is an angular error between the input axis of the accelerometer and the direction of the centripetal acceleration vector. In this paper, the distance between the center of the sub-turntable and the sensitive center of the accelerometer is defined as the installation error of radius *θ*_1_, and the angle between the input axis of the accelerometer, and the direction of the centripetal acceleration vector is defined as installation error of angle.

**Figure 2:**
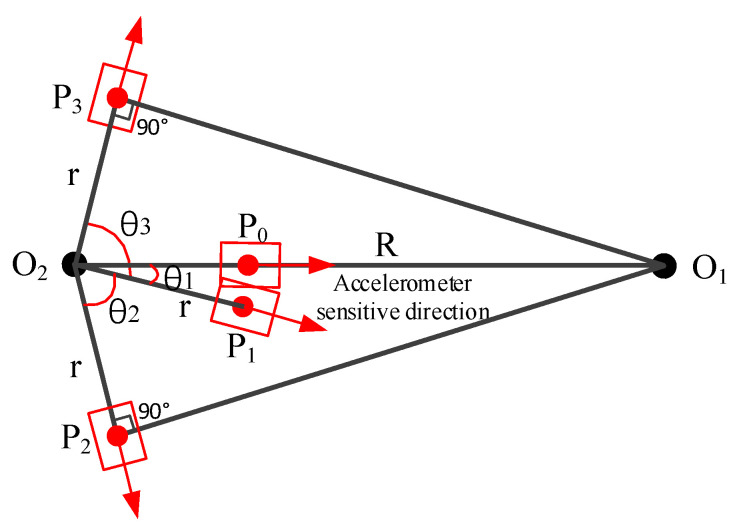
Geometric diagram in consideration of installation errors.

In consideration of the installation errors, the geometric diagram of the installation is shown in Figure 2, where *P*_1_ is the actual position of initial state, and *P*_0_ is the target position that the accelerometer needs to be adjusted to. The input axis of the measured accelerometer is not aligned with *O*_1_*O*_2_, and its angle is *θ*_1_. The installation error of radius *r* is the distance between *P*_1_ and *O*_2_.

### 2.3. Model of Our Proposed Calibration Method

In our calibration, the position of sample on the sub-turntable should be rotated from *P*_1_ to *P*_0_. The sub-turntable is rotated counterclockwise with an angle *θ*_1_ to make the input axis of the measured accelerometer coincide with the direction of the centripetal acceleration vector. In this case, the actual input acceleration is as follows:

(2) $$a=\omega^{2}\times \left(R-r\right)$$

where *r* is the distance between the sensitive center of the measured accelerometer *P*_0_ and the rotation center *O*_2_ whose unit is m.

Then, the sub-turntable is rotated clockwise with an angle *θ*_2_ to position, and the angle between *O*_2_*P*_2_ and *O*_1_*P*_2_ is 90°, as shown in Figure 2. At this position *P*_2_, the input axis of the accelerometer is perpendicular to the direction of the centripetal acceleration vector, and the amplitude of centripetal acceleration imposed on the input axis of the accelerometer is zero. Similarly, the sub-turntable is rotated counterclockwise with an angle *θ*_3_ to make the angle between *O*_2_*P*_3_ and *O*_1_*P*_3_ 90°. According to the geometric relationship, the following equation can be obtained:

(3) $$\theta_{3}-\theta_{1}=\theta_{2}+\theta_{1}$$

According to Equation (3), the installation error of angle *θ*_1_ and the installation error of radius *r* can be obtained as follows:

(4) $$\theta_{1}=\left(\theta_{3}-\theta_{2}\right)/2$$

(5) $$r=R\times \cos\left(\theta_{2}+\theta_{1}\right)=R\times \cos\left(\frac{\theta_{2}+\theta_{3}}{2}\right)$$

The sub-turntable is rotated counterclockwise with an angle *θ*_1_ to make the input axis of the measured accelerometer collinear with the direction of the centripetal acceleration vector. Then, the actual static radius of the accelerometer *R*_0_ can be obtained as follows:

(6) $$R_{0}=R-r=R\times \left[1-\cos\left(\frac{\theta_{2}+\theta_{3}}{2}\right)\right]$$

Based on the calibrated static radius *R*_0_, the calibrated input acceleration of the measured accelerometer *a*_0_ can be obtained as follows:

(7) $$a_{0}=a\times \frac{R_{0}}{R}=a\times \left[1-\cos\left(\frac{\theta_{2}+\theta_{3}}{2}\right)\right]$$

## 3. Measurement Method of the Calibration

### 3.1. Procedure of Calibration

**Figure 3:**
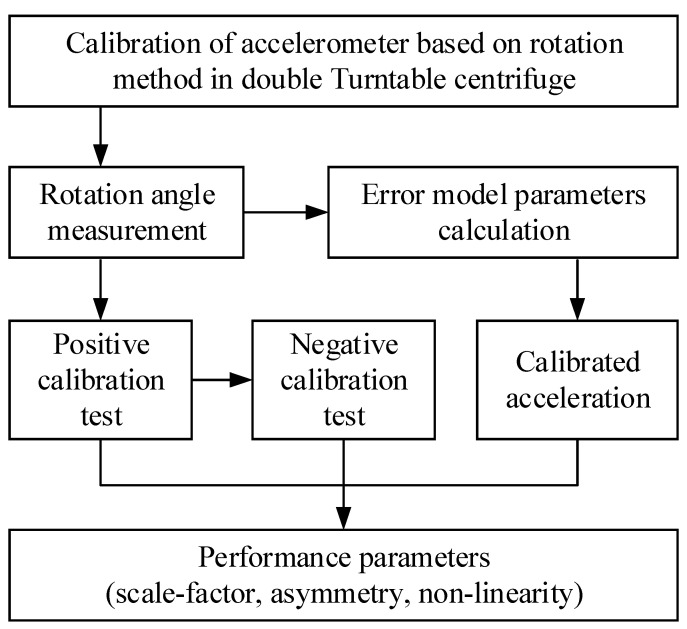
Calibration flow of accelerometer.

The test procedure of calibrating the accelerometer based on our proposed rotation method in the double turntable centrifuge is shown in Figure 3.

### 3.2. Theory of Measuring the Rotation Angle

In our proposed method, the acquisition of rotation angles *θ*_2_ and *θ*_3_ is crucial. Measuring these angles precisely is a key step for obtaining the installation error of angle *θ*_1_ and installation error of radius *r*.

Theoretically, the accelerometer can only detect the acceleration imposed on its input axis, but the cross-axis sensitivity leads to output error. In consideration of the second-order nonlinearity and cross-axis error, the mathematical model of accelerometer output can be expressed as:

(8) $$U=K_{0}+K_{1}\times a_{i}+K_{2}\times a_{i}{}^{2}+\lambda_{0}\times a_{0}+\lambda_{p}\times a_{p}$$

where U is the output of accelerometer, *K*_0_ is bias, *K*_1_ is the scale factor, *K*_2_ is the second-order nonlinear coefficient, *λ*_0_ and *λ_p_* are the cross-axis sensitivity of the output axis and pendulum axis, respectively, and *a_i_*, *a*_0_ and *a_p_* are the accelerations imposed on the input axis, output axis and pendulum axis of the accelerometer, respectively.

When the main turntable is rotating steadily, the sub-turntable is rotated with an angle to position where the output U_1_ is equal to the static output *U*_0_. Then, Equation (9) can be obtained as follows:

(9) $$K_{0}+\lambda_{0}\times 1g=K_{0}+K_{1}\times a_{i}+K_{2}\times a_{i}{}^{2}+\lambda_{0}\times 1g+\lambda_{p}\times a_{p}$$

When the acceleration imposed on the pendulum axis *a_p_* and the sensitivity of the cross axis *λ_p_* are both small, the value of *λ_p_* × *a_p_* is close to zero and can be ignored. Therefore, the acceleration imposed on the input axis of the measured accelerometer *a_i_* is zero according to Equation (9), which indicates that the input axis of the accelerometer is perpendicular to the direction of the centripetal acceleration vector.

### 3.3. Procedure of Measuring the Rotation Angle

The experimental procedure of measuring the rotation angles of *θ*_2_ and *θ*_3_ based on our rotation method in a double turntable centrifuge is as follows:

(1) After the precision centrifuge is powered on and reset, the measured accelerometer is installed and fixed on the sub-turntable.

(2) The accelerometer is powered on. After the sample is thermally stable, its output is recorded for 30 s, and the average value is calculated as U_0_.

(3) The main turntable is rotated steadily with an angular rate *ω*.

(4) The sub-turntable is rotated clockwise with an angle, and the output of the sample at this position is recorded, and the data are compared with U_0_.

(5) The clockwise rotation angle of the sub-turntable is adjusted until the average value of the output is equal to U_0_, and the rotation angle of the sub-turntable at this position is recorded as *θ*_2_.

(6) Similarly, the sub-turntable is rotated counterclockwise with an angle, and the output of the sample at this position is recorded, and the data are compared with U_0_.

(7) The counterclockwise rotation angle of the sub-turntable is adjusted until the average value of the output is equal to U_0_, and the rotation angle of the sub-turntable at this position is recorded as *θ*_3._

(8) Based on values of the rotation angles *θ*_2_ and *θ*_3_, the installation error of angle *θ*_1_ and installation error of radius *r* can be calculated according to Equations (4) and (5).

(9) The calibrated input acceleration is obtained subsequently. The calibration of the accelerometer can then be implemented.

## 4. Calibration Results and Discussion

**Figure 4:**
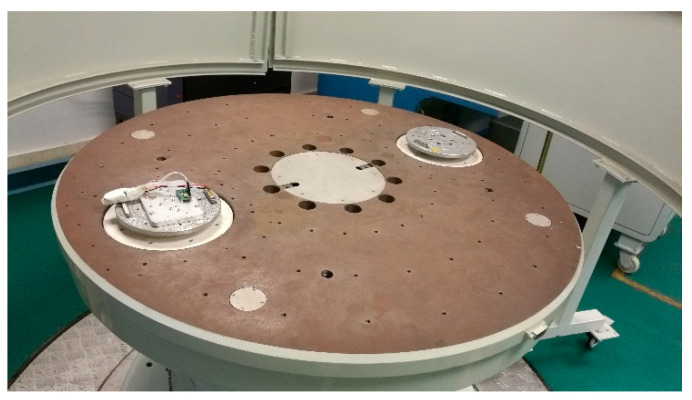
Installation drawing of test sample.

MEMS technology is developing rapidly, and the MEMS accelerometer is widely used in fields of industry and military for its small volume and high performance. Therefore, the MEMS accelerometer was selected to verify our calibration method. The sample is a silicon-based comb accelerometer, and the range of the high-precision accelerometer is ±30 g. The sample and its installation on the double turntable centrifuge are shown in Figure 4.

### 4.1. Measurement of Calibration Error Model Parameters

In the procedure of measuring the rotation angles *θ*_2_ and *θ*_3_, the centripetal acceleration of the main turntable during operation should be a small value that can reduce the measurement error caused by the lateral acceleration, and the acceleration in this measurement was 0.5 g.

The accelerometer was installed and fixed on the sub-turntable of the double turntable centrifuge, and the rotation angles *θ*_2_ and *θ*_3_ can be obtained by coarse and fine adjustment. In this measurement, *θ*_2_ and *θ*_3_ were 86.9020° and 89.0205°, respectively.

To verify the accuracy of rotation angles, a verification experiment was designed. If the direction of the centripetal acceleration vector is perpendicular to the input axis of the accelerometer and the influence of lateral acceleration is small enough, the output of accelerometer does not change with the different centripetal accelerations. Firstly, the sub-turntable was rotated clockwise with an angle of *θ*_2_ = 86.9020°, and the centripetal acceleration of the main turntable was set to 5, 4, 3, 2, 1 and 0 g in sequence. The output of the sample within 10 s at each acceleration was recorded, and the average values are shown in Table 1. The experimental data show that the output of the measured accelerometer remains approximately constant when different centripetal acceleration was imposed. Thus, the verification results of rotation angles demonstrate that the error caused by the lateral acceleration can be ignored, and our measured rotation angles are accurate.

Based on the measured rotation angles *θ*_2_, *θ*_3_ and Equation (4), the installation error of angle was *θ*_1_ = (*θ*_3_ *− θ*_2_)/2 = (89.0205° − 86.9020°)/2 = 1.05925°. Therefore, the sub-turntable should be rotated counterclockwise with an angle of 1.05925° to the position *P*_0_, where the input axis of sample is parallel to the direction of the centripetal acceleration vector. Based on the measured rotation angles *θ*_2_, *θ*_3_ and Equation (5), the installation error of radius was *r* = *R* × cos [(*θ*_2_ + *θ*_3_)/2] = 0.4 m × cos [(86.9020° + 89.0205°)/2] = 0.014230 m.

### 4.2. Calibration Test of MEMS Accelerometer

**Figure 5:**
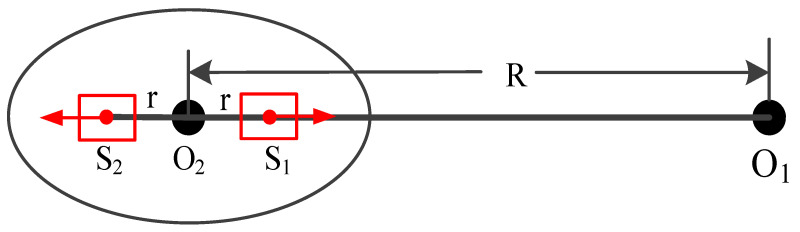
Geometric diagram of calibration test for accelerometer.

According to the measured installation error of angle *θ*_1_ and installation error of radius *r*, the calibration test of the MEMS accelerometer can be implemented. And the scale factor, asymmetry and nonlinearity of the accelerometer can be measured and calibrated. In the calibration measurement, there are positive calibration test and negative calibration test, and the geometric diagram is shown in Figure 5.

Firstly, negative calibration test was performed. The sub-turntable was rotated counterclockwise with an angle of 1.05925° to the position *S*_1_, where the input axis of sample was anti-parallel to the direction of the centripetal acceleration vector. Therefore, the static radius in the negative calibration test was *R*_–_ = *R* − *r* = 0.4 − 0.014230 = 0.385770 m, and the calibration coefficient for reverse input acceleration was 0.385770/0.4 = 0.964425. The acceleration of the main turntable was rotating with centripetal acceleration of 0, −1, −2, −5, −10, −15, −20, −25 and −30 g in sequence. Meanwhile, the output voltages of the accelerometer were recorded, and the results of negative calibration test are shown in Table 2

Secondly, the positive calibration test was performed similarly. The sub-turntable was then rotated clockwise with an angle of 180° to the position *S_2_*, where the input axis of sample was parallel to the direction of the centripetal acceleration vector. Therefore, the static radius in the positive calibration test was *R_+_ = R* *+ r =* 0.4 m + 0.014230 m = 0.414230 m, and the calibration coefficient for forward input acceleration was 0.414230/0.4 *=* 1.035575. The acceleration of the main turntable rotated with centripetal acceleration of 0, 1, 2, 5, 10, 15, 20, 25 and 30 g in sequence. Meanwhile, the output voltages of the accelerometer were recorded, and the results of the positive calibration test are shown in Table 3.

**Figure 6:**
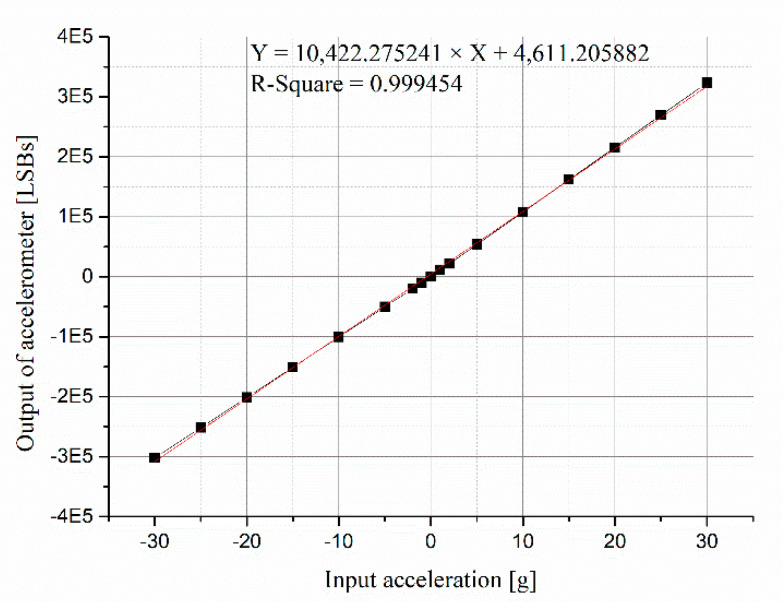
Linear fitting of scale factor before calibrating input accelerations.

**Figure 7:**
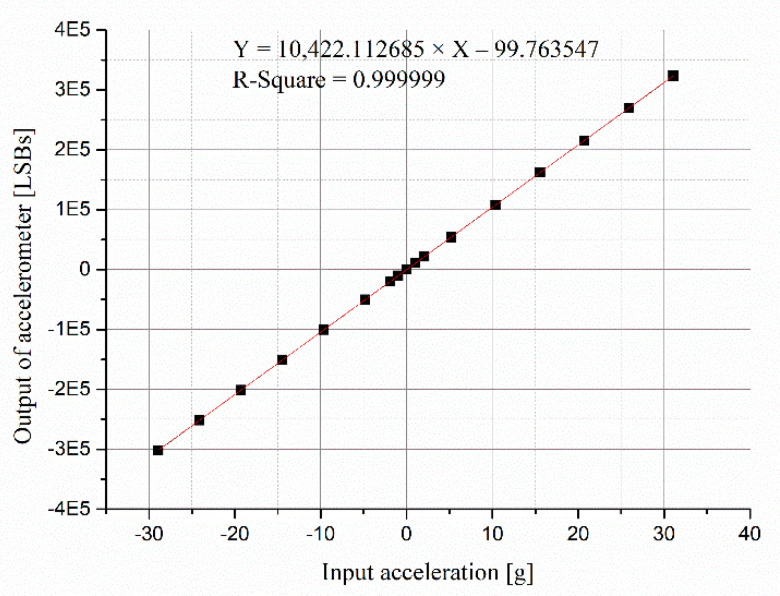
Linear fitting of scale factor after calibrating input accelerations.

The output of the accelerometer was analyzed with the input acceleration before and after calibration. The linear fitting curves of the scale factor before and after calibrating the input acceleration are shown in Figure 6 and Figure 7, respectively. The data of the horizontal axis in Figure 6 are accelerations of centrifuge, and the data of the horizontal axis in Figure 7 are calibrated input accelerations.

### 4.3. Discussion

According to the data processing and analysis method [25], the performance of an accelerometer can be calculated, including the scale factor of positive-scale *K*_1+_, the scale factor of negative-scale *K*_1−_, the scale factor of full-scale *K*_1_, asymmetry and nonlinearity. The results before and after calibrating input accelerations are compared in Table 4.

It can be seen that the scale factors of full-scale *K*_1_ of the two cases are almost equal. However, the difference of *K*_1+_ and *K*_1−_ before calibration is large, and the nonlinearity is up to 9957 ppm. This is because the input acceleration is not calibrated, which seriously exaggerates the nonlinearity of the sample. Through calibrating the input acceleration, the actual nonlinearity is 537 ppm. These results clearly show that the asymmetry and nonlinearity of the measured accelerometer are obviously distorted if the input acceleration is not calibrated. Therefore, when the precision centrifuge is used for measuring the accelerometer, the acceleration imposed on accelerometer must be calibrated, and our proposed method can greatly reduce the calibration error.

In addition, the results using our proposed method are consistent with those using the back-calculation method. The compared results are shown in Table 5. It can be seen that the scale factors, asymmetry and nonlinearity measured by the two methods are highly consistent, which demonstrates the effectiveness of our proposed method.

In order to confirm the effectiveness of our proposed method, an experiment was designed. The same measured accelerometer was deliberately installed at different positions on the sub-turntable, and the measured results at different positions were compared. If the consistency of the results is good, the effectiveness of our proposed method can be confirmed.

Therefore, the sample was disassembled and reinstalled. The installation errors of angle and radius were measured again, and the performance of the accelerometer at the different positions were obtained. The test results at the two different positions are compared in Table 6. There are obvious differences in the installation error of angle and the installation error of radius at position 1 and position 2. This is because the sample was deliberately installed at different positions on the sub-turntable. Nevertheless, the performance of our proposed method at different installation positions shows a high degree of consistency which is smaller than 100 ppm, proving the effectiveness of our proposed method.

## 5. Conclusions

To solve installation errors in the calibration of the accelerometer, this paper proposes a calibration method for accelerometer using a double turntable centrifuge. This method can eliminate the effect of installation errors of angle and radius, and the static radius can be measured directly based on our established model. The input acceleration can be calibrated using our proposed method, and the scale factor, nonlinearity, asymmetry of the accelerometer can be accurately measured.

The MEMS accelerometer was used to verify this calibration method. Our proposed method can greatly reduce the calibration error, and the comparative results of different installation positions and different methods all verified the effectiveness of this method. The calibration process of our proposed method requires only centrifuge and one installation, and so it can simplify the calibration procedure and improve the calibration accuracy. This method can be used in the calibration of the accelerometer, and it reveals a high potential of engineering applicability.

## Figures and Tables

**Table 1:** Verification data of rotation angles.

Lateral Acceleration (g)	Output of Accelerometer (mg)
5	−60.7116
4	−60.7106
3	−60.7094
2	−60.7100
1	−60.6948
0	−60.7131

**Table 2:** Data of negative calibration test.

Acceleration of Centrifuge (g)	Calibrated Input Acceleration (g)	Output of Accelerometer (LSBs)
0	0	0
−1	−0.964425	−10,058.2
−2	−1.928849	−20,111.8
−5	−4.822123	−50,294.5
−10	−9.644246	−100,598.1
−15	−14.466369	−150,900.9
−20	−19.288492	−201,214.5
−25	−24.110615	−251,524.6
−30	−28.932738	−301,832.3

**Table 3:** Data of positive calibration test.

Acceleration of Centrifuge (g)	Calibrated Input Acceleration (g)	Output of Accelerometer (LSBs)
0	0	0
1	1.035575	10,801.9
2	2.071151	21,604.2
5	5.177877	53,998.7
10	10.355754	107,954.5
15	15.533631	161,871.5
20	20.711508	215,757.5
25	25.889385	269,586.4
30	31.067262	323,350.7

**Table 4:** Test results of MEMS accelerometer before and after calibration.

Parameter	Before Calibration	After Calibration
*K*_1+_ (LSB/g)	10,780.33	10,409.99
*K*_1−_ (LSB/g)	10,061.18	10,432.32
*K*_1_ (LSB/g)	10,422.28	10,422.11
asymmetry (ppm)	69,011	2142
nonlinearity (ppm)	9957	537

**Table 5:** Test results of MEMS accelerometer using different calibration method.

Parameter	Our Method	Back-Calculation Method
*K*_1+_ (LSB/g)	10,409.99	10,409.90
*K*_1−_ (LSB/g)	10,432.32	10,434.28
*K*_1_ (LSB/g)	10,422.11	10,423.08
asymmetry (ppm)	2142	2339
nonlinearity (ppm)	537	565

**Table 6:** Measurement results at different positions after calibration.

Parameter	Position 1	Position 2
*θ*_2_	86.9020°	89.5128°
*θ*_3_	89.0205°	90.4210°
*θ*_1_	1.05925°	0.4541°
*r* (m)	0.01423016	0.00023108
*K*_1_ (LSB/g)	10,422.11	10,421.67
asymmetry (ppm)	2142	2118
nonlinearity (ppm)	538	530
